# 3D-printed in vitro models of Stanford type B aortic dissection: A scoping review

**DOI:** 10.1016/j.jvscit.2025.101987

**Published:** 2025-09-19

**Authors:** Matthias Niklas Hagedorn, Marcello Mächerle, Roger Karl, C. Soeren Bergt, Dittmar Böckler, Sandy Engelhardt, Katrin Meisenbacher

**Affiliations:** a Department of Vascular and Endovascular Surgery, Heidelberg University Hospital, Heidelberg, Germany; b Department of Cardiac Surgery, Heidelberg University Hospital, Heidelberg, Germany; c Department of Internal Medicine III, Department of Cardiology, Heidelberg University Hospital, Heidelberg, Germany

**Keywords:** 3D-printing, Type B aortic dissection, Simulation, Perfusion model, Three-dimensional phantom

## Abstract

Patient-specific three-dimensional-printed phantoms have emerged as valuable tools for simulating Stanford type B aortic dissections in vitro, enabling detailed studies of dissection morphology, hemodynamics, and interventional techniques under controlled, anatomically realistic conditions. Despite their potential, current methodologies remain heterogeneous and lack standardization. This scoping review, compliant with the PRISMA guidelines, systematically evaluated literature describing additive-manufactured flexible aortic phantoms specifically for pulsatile flow experiments or endovascular procedures. From an initial pool of 120 publications, five studies met the inclusion criteria, all using clinical imaging data and PolyJet-technology with flexible photopolymers. Four studies used full-scale models to simulate hemodynamics or thoracic endovascular aortic repair, and one investigated imaging properties in smaller segments. Although these phantoms reliably replicate dissection anatomy and flow patterns, widespread adoption is constrained by resource demands, simplified wall mechanics, exclusion of smaller vessel branches, and variable fabrication methods. Additional limitations include material durability and single-use designs. Standardizing fabrication protocols and developing advanced biomimetic materials could significantly enhance the physiological accuracy, reproducibility, and practicality of these models. Patient-specific three-dimensional-printed type B aortic dissections phantoms thus represent potentially valuable tools for improving surgical training, procedural rehearsal, morphological insights, and device innovation, ultimately bridging benchtop simulations and clinical practice.

Thoracic endovascular aortic repair (TEVAR) is the standard treatment for complicated Stanford type B aortic dissections (TBADs) and is increasingly considered for selected patients with uncomplicated disease.^1^, ^2^, ^3^, ^4^, ^5^ Recent studies have demonstrated favorable remodeling, improved aortic-related survival, and false lumen thrombosis in patients undergoing subacute TEVAR in uncomplicated cases.^4^, ^5^, ^6^ In addition, TEVAR is also used in the management of aneurysmal degeneration in the chronic phase of TBAD.^7^

However, the procedure itself remains technically demanding, particularly in patients with complex aortic anatomies, highly mobile intimal flaps in the acute and subacute phase, or narrow true lumens in the chronic phase where flap mobility is reduced. The biomechanical fragility of the dissected aortic wall, together with the challenge of secure wire placement, continues to pose challenges for durable endovascular outcomes despite the routine use of adjunctive imaging modalities such as intravascular ultrasound, transesophageal echocardiography, or stepwise angiography.^8^, ^9^, ^10^, ^11^

The increasing complexity of devices and the clinical diversity of TBAD patient subtypes, combined with restricted opportunities for structured surgical training owing to the increasing workload, cost pressures, and safety considerations, created a need for robust, standardized, and anatomically realistic preclinical models.^12^

In this context, three-dimensional (3D)-printed in vitro models have emerged as potentially promising tools for experimental evaluation and procedural simulation.^13^ Although silicone-based phantoms remain valuable for training and generic hemodynamic studies, 3D printing provides unique advantages by enabling replication of patient-specific vascular geometries, rapid reconfiguration of morphological details (eg, entry and re-entry tears), and access to a range of materials with adjustable mechanical properties. These features facilitate the reproduction of pulsatile flow conditions and allow detailed hemodynamic assessment using 4D-flow magnetic resonance imaging (MRI), fluid-structure interaction (FSI), or Doppler ultrasound examination.^14^ Furthermore, fluoroscopy-guided simulation of TEVAR procedures in these phantoms may support endograft testing, procedural planning, and advanced training scenarios.^14^

Despite these promising applications, the current methodological landscape of 3D-printed TBAD models is heterogeneous. Published studies vary in imaging source and segmentation methods, printing resolution, material properties (eg, TangoPlus vs Agilus30), and the physiological realism of flow circuits. Additionally, there is limited consensus regarding the extent to which these models accurately replicate aortic biomechanics or dynamic wall behavior. These inconsistencies complicate direct comparison across studies and limit broader clinical translation.

Our previously published proof-of-concept study demonstrated the feasibility of performing fluoroscopy-guided TEVAR in a perfused, patient-specific, 3D-printed TBAD model.^14^ Building on this work, the present scoping review aims to systematically identify, characterize, and contextualize existing in vitro TBAD models produced using 3D printing technologies. Special emphasis is placed on methodological standards, mechanical fidelity, and the translational potential of these models for device development, morphological hypothesis testing, and endovascular training.

## Methods

We focus on models created by additive manufacturing that are used in benchtop simulations. Models cast from a 3D-printed mold but made of silicone, as well as nonprinted models, were excluded. Only studies that explicitly use 3D printing to fabricate TBAD phantoms for experimental purposes were considered.

A systematic literature search was performed in PubMed (MEDLINE) using the terms “aortic dissection,” “descending aortic dissection,” “type B aortic dissection,” and “dissection” in combination with “3D printing,” “three-dimensional printing,” “3D phantom,” or “three-dimensional phantom.” The search was performed on May 25, 2025, in accordance with the PRISMA guidelines for scoping reviews.^15^ In addition to the included full-text articles, reference lists were screened for further relevant publications without time restriction. The screening was carried out independently by two authors (M.H., M.M.).

### Inclusion and exclusion criteria

We included TBAD phantoms, models created by 3D printing techniques, and in vitro or benchtop usage (eg, imaging, flow experiments, device deployment). We excluded clinical case reports without phantoms, purely computational studies, commercial static models, 3D-printed casts for silicone model fabrication, educational models for illustrative purposes only, 3D-printed models for open repair planning, and models to produce templates for extracorporeal modification of stent grafts during complex endovascular procedures.

For each included study, data on imaging source (eg, computed tomography angiography [CTA]), segmentation method, printing technology, materials, whether models were patient specific, experimental setup (flow systems, imaging or interventional procedures), and reported outcomes were extracted. Key findings and limitations from each study were synthesized qualitatively.

## Results

**Fig 1:**
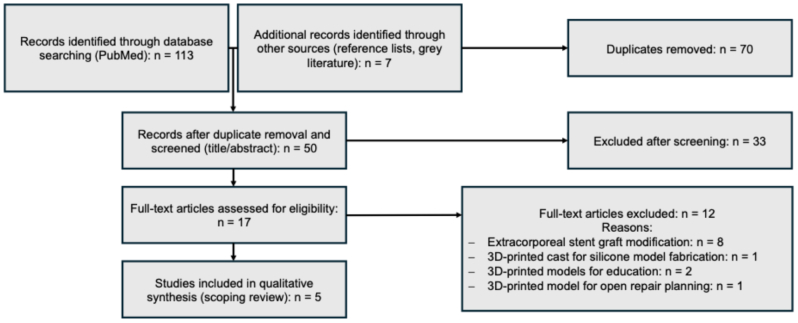
Flow diagram of study selection conducted in accordance with THE PRISMA guidelines.

A total of 113 records were initially identified via PubMed, with an additional 7 records obtained through gray literature and reference lists. After removing duplicates (n = 70), 50 records were screened based on title and abstract, resulting in 17 articles undergoing full-text review (Fig 1). Finally, five relevant studies meeting the inclusion criteria in this qualitative synthesis were included (Table). Notably, these five studies originated exclusively from three distinct research groups: Stanford University, Heidelberg University Hospital, and Curtin University, Perth.

**Table:** Overview of included studies on three-dimensional (3D)-printed type B aortic dissection (*TBAD*) models

Study (first author)	Research group	Patient specific?	Material and printing method	Setup and application	Highlights	Limitations
Zimmermann et al. (2023)^16^	Stanford	Yes (CTA based)	Agilus30 resin (PolyJet), waterproof coating	Pulsatile flow loop; 4D-flow MRI and pressure measurement; FSI comparison	Realistic true vs false lumen flow profiles; entry/exit tear size effects on hemodynamics; close correlation between MRI and FSI	Single-patient model limits generalizability; uniform wall/flap thickness and elasticity; no in vivo prestress simulation; highly simplified model without visceral branches
Zimmermann et al. (2021)^17^	Stanford	Yes (CTA based)	Agilus30 resin (PolyJet), variable stiffness	Pulsatile MRI flow loop; 4D-flow MRI measurements	Developed compliant aortic model; showed increased wall stiffness raises peak pressure and PWV; better in vivo hemodynamic fidelity	Only one geometry; rigid comparison material did not match real aortic stiffness; MRI temporal resolution blurred results
Zimmermann et al. (2021)^13^	Stanford	Yes (CTA based)	Agilus30 resin (PolyJet)	4D-flow MRI in vitro vs FSI simulation (validation study)	Demonstrated concordance of flows, velocities, and pressure curves between experiment and FSI; validated simulation approach	Brief conference report; single model case; no further parameter or geometry studies
Mohl et al. (2025)^14^	Heidelberg	Yes (CTA based)	TangoPlus resin (PolyJet)	Full-scale model in pulsatile loop; fluoroscopy-guided TEVAR simulation	First TEVAR in 3D-printed TBAD phantom under clinical conditions; physiological pressures and flows; true/false lumen dynamics; high anatomical accuracy with visualization of all aortic branches	High production effort (thin intima printing); material fatigue (cracks/leaks after use); limited model lifespan; small side branches and peripheral resistance omitted
Wu et al. (2021)^18^	Curtin University	Partial (25-mm segment)	Agilus A40/A50; Visijet CE-NT A30/A70 (PolyJet and MJP)	Static segment phantom; CT imaging (native + contrast)	Agilus A50 matched native aortic CT attenuation; Visijet A30 reproduced contrast uptake; Visijet A30 tensile strength ∼ aged aorta	Only short segment, not full anatomy; no flow or pressure simulation; findings limited to imaging/material properties; uncertain transferability to simulation models with full anatomy

*CTA,* Computed tomography angiography; *FSI,* fluid-structure interaction; *MRI,* magnetic resonance imaging; *PWV,* pulse wave velocity; *TEVAR,* thoracic endovascular aortic repair.

## Discussion

This scoping review demonstrates that 3D-printed TBAD models are already being used to investigate clinically relevant morphological and hemodynamic aspects. The establishment of patient-specific, physiologically perfused platforms may provide valuable complementary insights into the pathophysiological and anatomical mechanisms that are difficult to capture with current in vivo imaging or computational simulations alone.

### Approaches of different research groups

#### Stanford group

The Stanford team developed a series of simplified patient-specific TBAD phantoms without visceral branches printed in flexible Agilus resin (PolyJet-printer, Stratasys Medical) and integrated into pulsatile flow loops (cardiac output 4.3 L/min with 60 bpm and 110-120 mm Hg systolic blood pressure) for 4D-flow MRI and FSI studies. They first demonstrated in a synthetic aortic model derived from a healthy 50-year-old patient without dissection that increasing wall stiffness raises peak pressures, alters flow patterns, and increases pulse-wave velocity, highlighting the necessity of compliant vessel walls for realistic hemodynamic measurements.^17^ A subsequent comparison of in vitro 4D-flow MRI data with coupled FSI simulations showed excellent concordance,^13^ validating the numerical approach. Building on this work, Zimmermann et al^13^ perfused three variants of the same patient model - original, reduced entry tear diameter, and enlarged re-entry tear diameter—in an MRI-compatible loop, confirming consistent true and false lumen flow and pressure distributions; the Agilus30 resin exhibited compliance within the range reported for nondiseased human aorta, although this does not necessarily reflect the altered biomechanical properties of dissected aorta. Beyond this model-based work, the working group is investigating mathematical-theoretical models for flow analysis, but without using physical models. Bäumler et al^19^ extended these investigations numerically across acute to chronic stages, showing that reductions in false lumen jet velocity correlate with progressive lumen dilation.

Finally, Lan et al^20^ in 2023 validated a novel reduced continuum formulation for vascular FSI against in vitro MRI data obtained from a nondissected aorta, demonstrating its efficiency and accuracy, although its applicability to TBAD remains to be established.

#### The Heidelberg group

**Fig 2:**
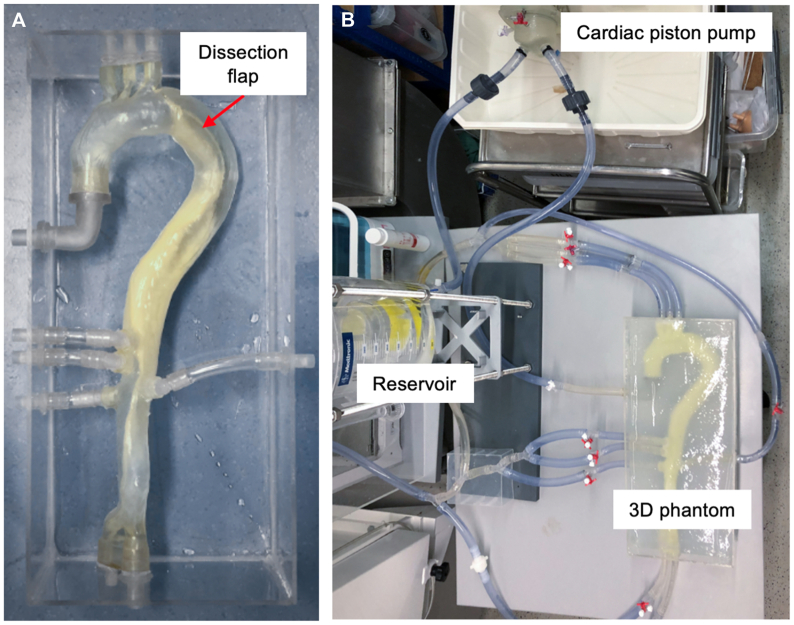
In vitro setup (Mohl et al) of a pulsatile-perfused 3D-printed aortic phantom with integrated dissection flap. **(A)** The phantom is mounted in a transparent fluid tank (*red arrow* indicates dissection flap). **(B)** It is connected via tubing to a fluid reservoir and cardiac piston pump, forming a closed-loop circuit that generates physiological pulsatile flow. *3D*, three-dimensional. The image originates from the authors' own work (Heidelberg group) and has not been published previously.

Our group concentrated on interventional simulation. A fully patient-specific TBAD phantom was fabricated, derived from CTA reconstruction and including all major branch vessels and a flexible dissection flap, using TangoPlus resin (PolyJet-printer, Stratasys Medical).^14^ The experimental setup is shown in Fig 2. When embedded in a closed pulsatile flow circuit (cardiac output ∼4 L/min), the phantom achieved physiological pressures (∼112 mm Hg systolic) and permitted successful fluoroscopy-guided deployment of a thoracic stent graft into the true lumen, with correct positioning confirmed angiographically and by postprocedure CTA.^14^ Flow measurements revealed realistic hemodynamics, with peak velocities of approximately 36 cm/s in the true lumen vs approximately 13 cm/s in the false lumen, as well as clear separation of flows and turbulent regions at entry and re-entry tears. This platform enabled a TEVAR intervention to be demonstrated in a 3D-printed TBAD model under near-realistic conditions. However, practical issues also emerged: owing to the very thin-walled intimal membrane, fabrication was complex, and the material showed signs of fatigue after just a few cycles—cracks and small leakages developed, limiting the model's lifespan. Furthermore, the model omitted smaller side branches and only simulated peripheral resistance in a simplified manner, which restricted its ability to accurately replicate the actual hemodynamics and the effects of the endovascular procedure.

#### The Curtin University group

The Curtin University group pursued a different approach, focusing on imaging properties and material characterization.^18^ A short, 25-mm segment of a dissected aorta was segmented from patient data and printed using various materials (Agilus in two hardness grades, Shore A40 and A50; as well as Visijet CE-NT in Shore A30 and A70). These short phantom segments were then scanned via CT, once empty and once filled with contrast agent in the lumen. The results showed that an Agilus model with Shore A50 closely matched the CT attenuation of a native aortic wall (∼80 Hounsfield units). In contrast, Visijet CE-NT A30 best replicated the contrast agent uptake of a real dissected aorta in CT (∼90 HU with contrast filling). Additionally, they demonstrated that the softest blend (Visijet A30) had a tensile strength comparable with aged aortic wall tissue.^18^ This study provided valuable insights into the radiological and mechanical validation of printing materials. However, it did not represent a fully functional flow model: no pulsatile pressure-flow conditions were simulated. Therefore, the transferability of these findings to larger, perfused models remains limited. Nonetheless, such material studies form the foundation for developing more realistic phantoms in terms of imaging and haptics in the future.

### The 3D printers used

The printing method used by all working groups, PolyJet printers, eg, Objet 500 Connex (Stratasys Medical, distributed by encee), has emerged as the preferred printing platform among multiple research groups.^13^^,^^14^^,^^16^, ^17^, ^18^ Its use enables rapid fabrication of identical models with different materials to closely simulate aortic wall properties.^14^ Because both the printer and its consumables are commercially available, the reproducibility of this approach is ensured.

### Materials used

The materials used have been either TangoPlus (Heidelberg group) or Agilus30 (Stanford group).^13^^,^^14^^,^^16^^,^^17^ Both exhibit a Shore hardness of approximately 30 A. The Shore hardness, expressed in Shore A units for soft materials on a standardized scale from 1 (very soft) to 100 (hard but still bendable), quantifies the relative softness or elasticity of polymeric materials and is commonly used to characterize elastomers in biomedical applications.

Despite similar nominal hardness, the two materials differ notably in their mechanical properties. Tear strength—defined as the material's resistance to the propagation of a tear once initiated—ranges from 2 to 4 kg/cm for TangoPlus and 5 to 7 kg/cm for Agilus30, suggesting that Agilus30 offers greater durability under mechanical stress. Tensile strength, which refers to the maximum stress a material can withstand while being stretched before rupture, is reported at 0.8 to 1.5 MPa for TangoPlus and 2.4 to 3.1 MPa for Agilus30, indicating greater structural integrity in the latter. Elongation at break—representing the material's capacity to stretch relative to its original length before failure—also differs (170%-240% for TangoPlus vs 220%-270% for Agilus30), reflecting Agilus30's superior flexibility and ductility. These differences may in part explain the crack formation and limited reusability observed in our model.^14^ However, the elastic capacity of TangoPlus far exceeds the stresses encountered at 120 mm Hg, suggesting that material fatigue from repetitive experimental loading and exposure to ultraviolet light is more likely responsible for the observed cracking. It is also possible that other research groups simply did not report material fatigue in their studies.

Cloonan et al^21^ (2013) further demonstrated that FullCure TangoPlus FLX930 exhibits ultimate tensile strengths, nonlinear elastic responses, and surface morphologies closely matching those of polydimethylsiloxane-based elastomers, thereby underscoring its suitability for pulse-wave ultrasound imaging and FSI simulations. More recently, several groups have proposed that combining materials (eg, TangoPlus with RGD450) can achieve mechanical properties nearly identical to those of native human aortic tissue, with Shore hardness values of approximately 30 A for dissected aortas and approximately 50 A for healthy aortas being the critical determinants.^22^, ^23^, ^24^

RGD450 (VeroWhitePlus, Stratasys) is a rigid photopolymer widely used in PolyJet printing. It exhibits a Shore D hardness of approximately 83, a tensile strength of 50 to 65 MPa, and an elongation at break of 10% to 25%, indicating high stiffness and low ductility. Shore D is a scale equivalent to Shore A, but used for harder polymers, such as rigid thermoplastics and structural photopolymers. Although not suitable for simulating soft tissue on its own, RGD450 is often used in combination with flexible materials to replicate layered vessel structures or to provide mechanical support in hybrid phantom designs.^24^

### Approaches to improve realism

The physiological realism of these models has been further enhanced by incorporating piston pumps simulating cardiac function. Several studies validated these pumps' capability to mimic realistic ventricular ejection patterns and hemodynamic conditions using water-glycerol mixtures as blood-mimicking fluids.^14^^,^^25^, ^26^, ^27^ In principle, such setups also allow the simulation of nonphysiological conditions—for instance, investigating the effects of systemic hypertension or intraoperative hypotension on the dissection membrane.^28^

### Financial resources

The principal cost drivers are high-performance 3D printers and materials. PolyJet systems commonly used in research (eg, Stratasys J7 series) cost between $55,000 and 330,000 plus maintenance. Flexible photopolymer cartridges (eg, TangoPlus FLX930) are approximately $935 per liter, and support materials (eg, SUP706 B) approximately $285 per kilogram; several kilograms may be required per model. Additional expenses include cleaning solutions, water, and servicing.

Specialized simulation infrastructure adds further costs. A pulsatile cardiac pump (eg, ViVitro SuperPump) is $33,000; flow sensors ($4730) and pressure transducers are essential for hemodynamic accuracy. Introducer sheaths cost approximately $275 each, and single-use consumables such as contrast, guidewires, and catheters add $550 to 1100 per experiment. Endografts, when implanted, contribute $5500 to 16,500, depending on the manufacturer and region.

### Nonfinancial resource requirements

Model preparation requires approximately 52 to 54 hours of active work. Segmentation (8 hours), postprocessing (8 hours), and adapter design (16 hours) constitute the digital phase. Printing takes 8 to 12 hours, followed by support removal over approximately 10 days (∼1 hour daily). Assembly requires 4 hours, but adhesive and silicone curing extend the process to approximately 48 hours. Experimental runs, including calibration and simulation, add 6 to 8 hours. Overall preparation typically spans 10 to 12 calendar days.

### Group-specific aspects and priorities

A comparative analysis of the included studies reveals distinct research priorities and methodological approaches among the respective groups, despite a shared focus on the development of 3D-printed TBAD phantoms using 3D printing technology.

The Stanford group focused primarily on the validation of hemodynamic measurements and numerical simulations with a simplified modell.^13^ In contrast, we (the Heidelberg group) prioritized interventional applicability and anatomical accuracy.^14^ They developed a full-scale, anatomically accurate phantom with a flexible dissection flap, enabling the first successful simulation of TEVAR under near-clinical conditions. The Curtin University group pursued a material-focused strategy, examining the imaging and mechanical properties of various print materials.^18^ Their static phantom segments allowed for the assessment of CT attenuation and tensile strength relative to native aortic tissue.

Collectively, these efforts illustrate the multifaceted nature of phantom design and highlight the need for integrated approaches that combine anatomical accuracy, imaging fidelity, mechanical realism, and dynamic functionality in future model systems.

### Limitations of the experimental phantom model

Current studies on 3D-printed TBAD models, although promising, are constrained by several methodological and practical limitations. Generalizability is decreased by the small number of patient-specific geometries investigated, and biomechanical realism is compromised by uniform vessel wall and flap thicknesses, as well as the absence of physiological prestress. The materials most used (eg, Agilus30, TangoPlus) do not reproduce the viscoelastic and remodeling properties of living tissue, leading to fatigue, cracking, and leakage with repeated use. In line with this, incomplete stent graft expansion in one model required balloon angioplasty—a maneuver usually avoided clinically owing to the risk of retrograde type A dissection—highlighting a major limitation for training purposes. Furthermore, peripheral branches and complex resistances are frequently omitted, limiting the capacity to replicate clinically relevant conditions such as distal malperfusion or false lumen thrombosis.

Although 3D-printed TBAD phantoms enable procedural rehearsal and assessment of acute technical feasibility, they cannot predict long-term outcomes such as aortic remodeling or end-organ perfusion. Limitations in branch inclusion, wall stress, and flap dynamics restrict their predictive value, positioning them as complementary to computational simulations better suited for modeling chronic processes. At the same time, computational models excel at predicting shear stress and remodeling, whereas physical phantoms uniquely allow hands-on assessment of device navigation, deployment accuracy, and acute hemodynamic responses. Both modalities should therefore be regarded as complementary rather than competing.

Differences in printing technologies and material properties complicate comparisons across studies and preclude standardized validation.

In addition to these methodological shortcomings, significant practical constraints persist. Model fabrication requires extensive infrastructure, considerable manual effort, and a substantial financial commitment. Secure integration of flexible phantoms into rigid flow circuits remains challenging and may result in leaks or stress concentrations, whereas moisture absorption and other material properties restrict reuse.^13^ Careful handling and degassing are essential to avoid experimental artifacts, particularly in imaging applications.^18^

Despite these limitations, the translational potential of 3D-printed TBAD phantoms is considerable. Short-term applications include device testing and procedural planning, and longer-term perspectives encompass surgical training and education.

Given the current resource intensity, 3D-printed TBAD phantoms are likely to remain restricted to specialized training environments (eg, advanced endovascular education or patient-specific case preparation). For broad educational use, established and cost-effective simulation tools are more appropriate until technological progress decreases cost and complexity. Nevertheless, considering the increasing constraints on hands-on training opportunities in clinical practice—driven by workload, financial pressure, and ethical considerations—such phantoms may provide a valuable and ethically acceptable environment to practice complex interventions under safe and reproducible conditions.^29^

Progress will depend on advances in materials with improved mechanical fidelity, the incorporation of prestressed wall conditions, and more realistic boundary representations. Embedding phantoms within compliant matrices, the adoption of standardized protocols, and the development of shared datasets will facilitate cross-validation and reproducibility. These improvements are essential to enhance reliability and ultimately establish 3D-printed TBAD models as potentially valuable translational adjuncts in vascular medicine.

### Opportunities and future applications

Beyond technical feasibility, perfused 3D-printed TBAD models offer a broad range of clinical and experimental opportunities. They allow systematic investigation of morphological parameters, such as the size, number, and location of entries and reentries, and their effect on true and false lumen perfusion before and after endovascular repair. Similarly, variations in membrane thickness—for example, to simulate chronic stiffening—can be studied to assess their impact on flow dynamics, wall stress, and treatment timing.

The platform also has the potential to facilitate comparative evaluation of different treatment strategies under controlled in vitro conditions. Quantitative flow and pressure data can be obtained at multiple sites, enabling a mechanistic understanding of procedure-related hemodynamic changes.

In addition, the models serve as valuable training tools for interventionalists in education and specialization, allowing hands-on simulation of fluoroscopy-guided procedures in a safe, repeatable environment. Importantly, costs and resource demands may be decreased substantially by leveraging existing infrastructure from related research fields, such as cardiovascular fluid dynamics or interventional imaging, thereby facilitating broader implementation beyond highly specialized centers. Furthermore, by correlating model-based data with intraoperative measurements and outcomes in real patients, this approach has the potential to bridge experimental insight and clinical translation in the management of aortic dissection.

Ultimately, if material and reproducibility challenges are addressed, these models could support the preclinical evaluation and regulatory testing of endovascular devices by offering standardized, anatomically realistic test conditions outside of animal or cadaveric settings.

## Conclusions

At this time, 3D-printed TBAD models offer valuable opportunities for studying complex aortic dissection dynamics and improving clinical procedures through realistic simulations. Although current limitations in material properties, anatomical fidelity, and practical handling remain significant challenges, ongoing technological advancements and collaborative standardization efforts are expected to enhance these models' clinical relevance substantially. Ultimately, these innovations promise to bridge the gap between experimental research and patient care, establishing 3D-printed phantoms as potentially valuable adjuncts in vascular surgery education, pathological understanding, and treatment optimization.

## Funding

The authors received financial support for the research of this project by the 10.13039/501100017515Heidelberger Stiftung Chirurgie.

## Disclosures

M.N.H. is part of the W. L. Gore & Associates Advisory Board, receives funding as part of a W. L. Gore & Associates Research Grant, and received a Travel Grant from W. L. Gore & Associates in August 2023, unrelated to this study. D.B. is consultant for W. L. Gore & Associates and Cook Medical and receives speaking honoraria, unrelated to this study. K.M. is part of the W. L. Gore & Associates Advisory Board and received a Travel Grant from W. L. Gore & Associates in August 2023, unrelated to this study.
